# Author Correction: Larval microbiota primes the *Drosophila* adult gustatory response

**DOI:** 10.1038/s41467-025-60716-2

**Published:** 2025-06-12

**Authors:** Martina Montanari, Gérard Manière, Martine Berthelot-Grosjean, Yves Dusabyinema, Benjamin Gillet, Yaël Grosjean, C. Léopold Kurz, Julien Royet

**Affiliations:** 1 Aix-Marseille Université, CNRS, IBDM, Marseille, France; 2 Centre des Sciences du Goût et de l’Alimentation, AgroSup Dijon, CNRS, INRAe, Université Bourgogne, F-21000 Dijon, France; 3 Institut de Génomique Fonctionnelle de Lyon, Ecole Normale Supérieure de Lyon, CNRS UMR5242, F-69007 Lyon, France

**Keywords:** Taste receptors, Neuroimmunology

Correction to: *Nature Communications* 10.1038/s41467-024-45532-4, published online 13 February 2024

Since the version of the article initially published, Fig. 4D has been amended, as seen below:

Original Fig. 4D:

**Figure:**
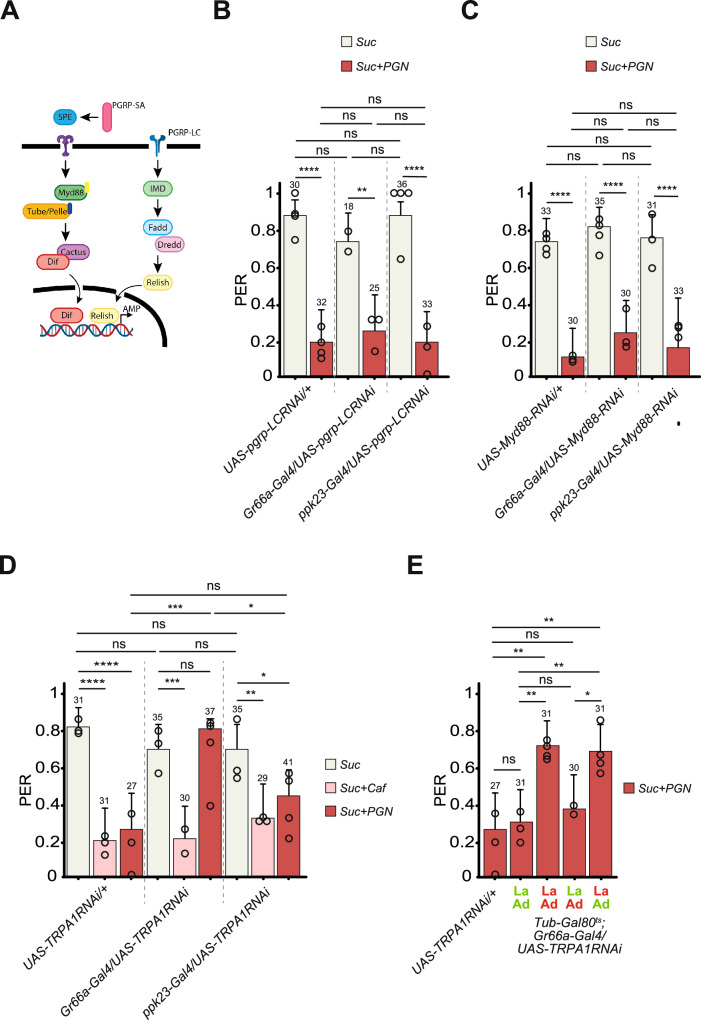

Corrected Fig. 4D:

**Figure:**
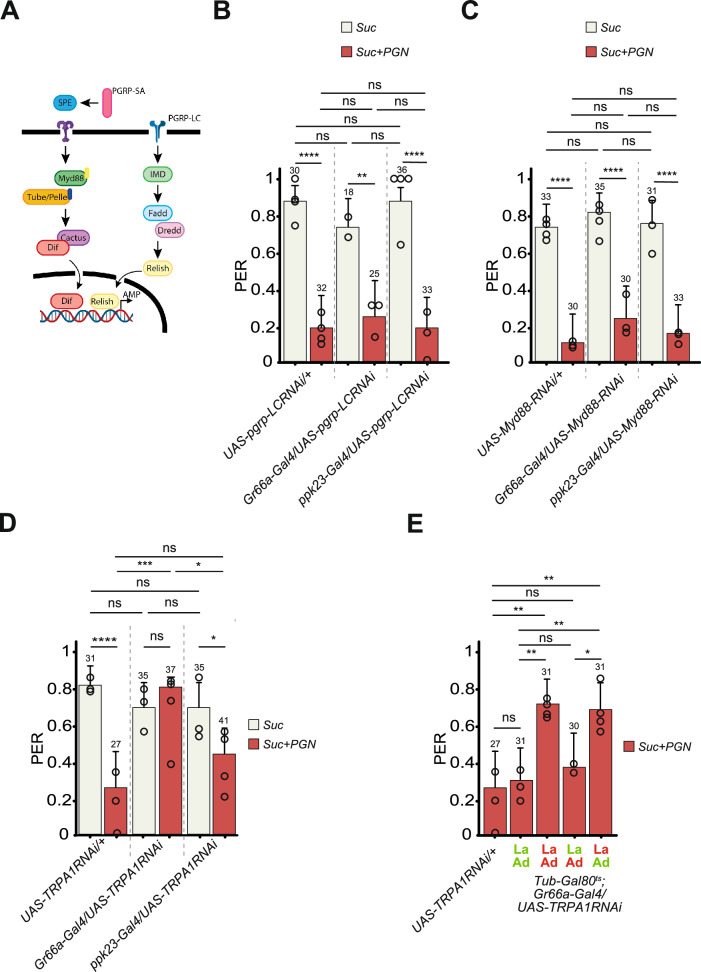

In this figure, we demonstrate using an RNA interference approach (Gr66a-Gal4/UAS-TrpA1-IR) that an aversion behavior (PER) toward a mixture of sucrose + peptidoglycan requires TrpA1 protein in a set of neurons (Gr66a+). Our control for these assays is the behavior toward sucrose, an attraction. In the same figure, we demonstrate that inactivating TrpA1 in another set of neurons (ppk23+) does not impair the aversion toward peptidoglycan. In addition, based on our Figures 1A–C and 2A–B, we aimed to illustrate the aversiveness of a benchmark repellent using caffeine. To do so, we tested the role of TrpA1 in the response to caffeine, as PER toward caffeine in TrpA1-RNAi flies had, to our knowledge, never been assessed before. Our results (Fig. 4D) showed that reducing TrpA1 activity via RNAi did not alter the flies’ aversion to caffeine—the response remained unchanged.

These data related to caffeine in the Fig. 4D are the ones we would like to remove.

While our results showing that reducing TrpA1 activity in larval Gr66a+ neurons blocks the response of adult flies to peptidoglycan are fully validated (Fig. 4E and current investigation in the laboratory) and in no way called into question, we later pursued the study of fly response to caffeine in the laboratory and demonstrated that TrpA1- mutants were no longer repelled by caffeine.

Intrigued by this result, we repeated the TrpA1-RNAi experiments in Gr66a neurons. This new and expanded series of RNAi experiments confirmed our results obtained with mutants i.e., TrpA1 is required in Gr66a+ neurons for the aversive response to PGN, but demonstrated that TrpA1 is also required in Gr66a+ neurons for the caffeine response.

We have no obvious explanations for the discrepancies between the two outcomes from what should be two identical experiments except that in the case of the published data, the absence of modifications following the treatment could be attributed to an absence of RNAi efficiency related to human mistakes as well as to an absence of involvement of the tested gene.

Since PER results are very reproducible and consistent for a given genotype from one experiment to the other and that we repeated the TrpA1 experiments with caffeine using independent mutants and RNAi and observed a modulation of the phenotype, we started to analyze the precise role of TrpA1 in caffeine sensing using series of mutants. As this is not the subject of this *Nature Communications* paper and since these data are not at all essential to the message (they were not specifically mentioned in the text), we propose to provide a new Fig. 4D without the data concerning TrpA1 and caffeine.

