# Supplementary material for: Larval microbiota primes the Drosophila adult gustatory response
Source: Nat Commun. 2024 Feb 13;15:1341. doi: 10.1038/s41467-024-45532-4 (PMC10864365; doi:10.1038/s41467-024-45532-4)
Supplement: Supplementary file 1 — Supplementary Information [file 41467_2024_45532_MOESM1_ESM.pdf]

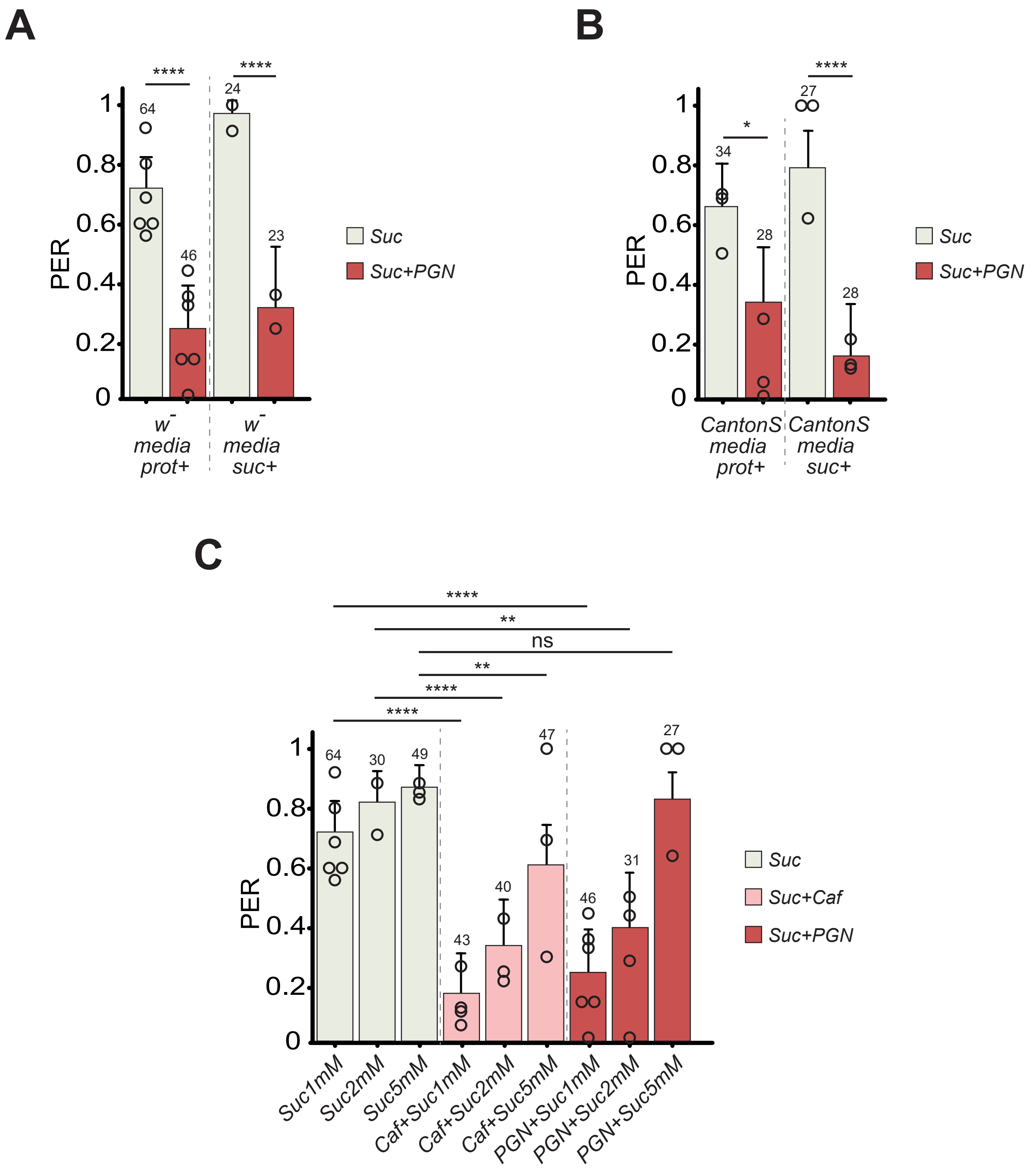

Fig.S1: Adult fly aversion to PGN is observed in different genetic backgrounds and in flies raised on different medias

(**A**) and (**B**), raising medium has no effect on PGN-induced PER suppression. PER index of *w-* (**A**) and CantonS (**B**) flies, raised on protein-rich (prot+) or sugar-rich (suc+) media, to control solutions of sucrose 1mM and to sucrose 1mM + PGN from *E. coli* K12 at 200µg/mL. The data for w- raised on media prot+ are the same as figure 1A, and those for CantonS raised on media prot+ are the same as figure 1C. (**C**) Sucrose concentrations above 5mM mask the aversive effect of PGN and caffeine. PER index of *w-* flies to increasing concentrations of sucrose, caffeine10mM + increasing concentration of sucrose and PGN from *E. coli* K12 at 200µg/mL + increasing concentrations of sucrose. For (**A**), (**B**) and (**C**) PER index is calculated as the percentage of flies tested that responded with a PER to the stimulation ± 95% CI. The number of tested flies (n) is indicated on top of each bar. For each condition, at least 3 groups with a minimum of 10 flies per group were used. ns indicates p>0.05, * indicates p<0.05, ** indicates p<0.01, **** indicates p<0.0001 two-sided Fisher Exact Test. Further details including raw data and exact p values can be found in the source data file.

A

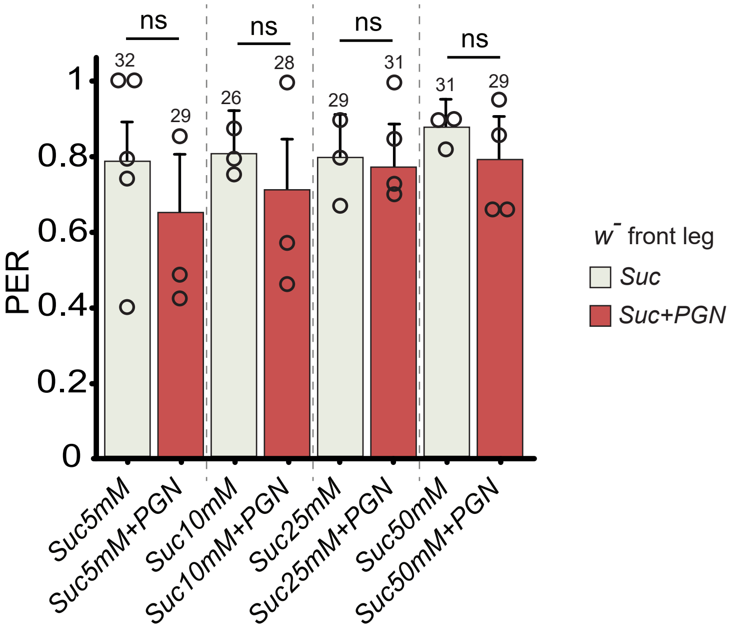

B

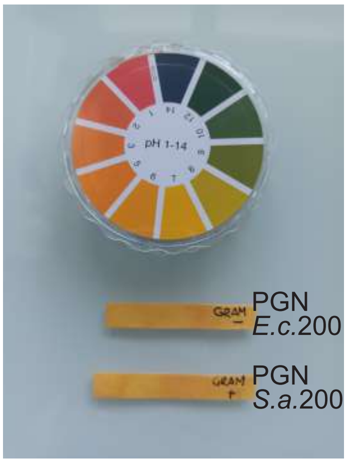

Fig.S2: PGN solutions are neutral

(**A**) PER index of *w*- flies upon stimulation of the front leg with increasing concentrations of sucrose or with PGN from *E. coli* K12 at 200µg/mL + increasing concentrations of sucrose. The numbers below the x-axis correspond to the sucrose final concentrations in mM. As 1mM sucrose does not trigger a PER robust and reproducible enough to qualify the animal as able to respond, this concentration was not used and we started with a sucrose concentration of 5mM. (**B**) PGN solutions from *E. coli* K12 (*E.c.*) and *S. aureus* (S.a.) at 200µg/mL are neutral. The pH of the two solutions was measured with pH test strips. For (**A**), PER index is calculated as the percentage of flies tested that responded with a PER to the stimulation ± 95% CI. The number of tested flies (n) is indicated on top of each bar. For each condition, at least 3 groups with a minimum of 10 flies per group were used. ns indicates p>0.05, two-sided Fisher Exact Test. Further details including raw data and exact p values can be found in the source data file.

A

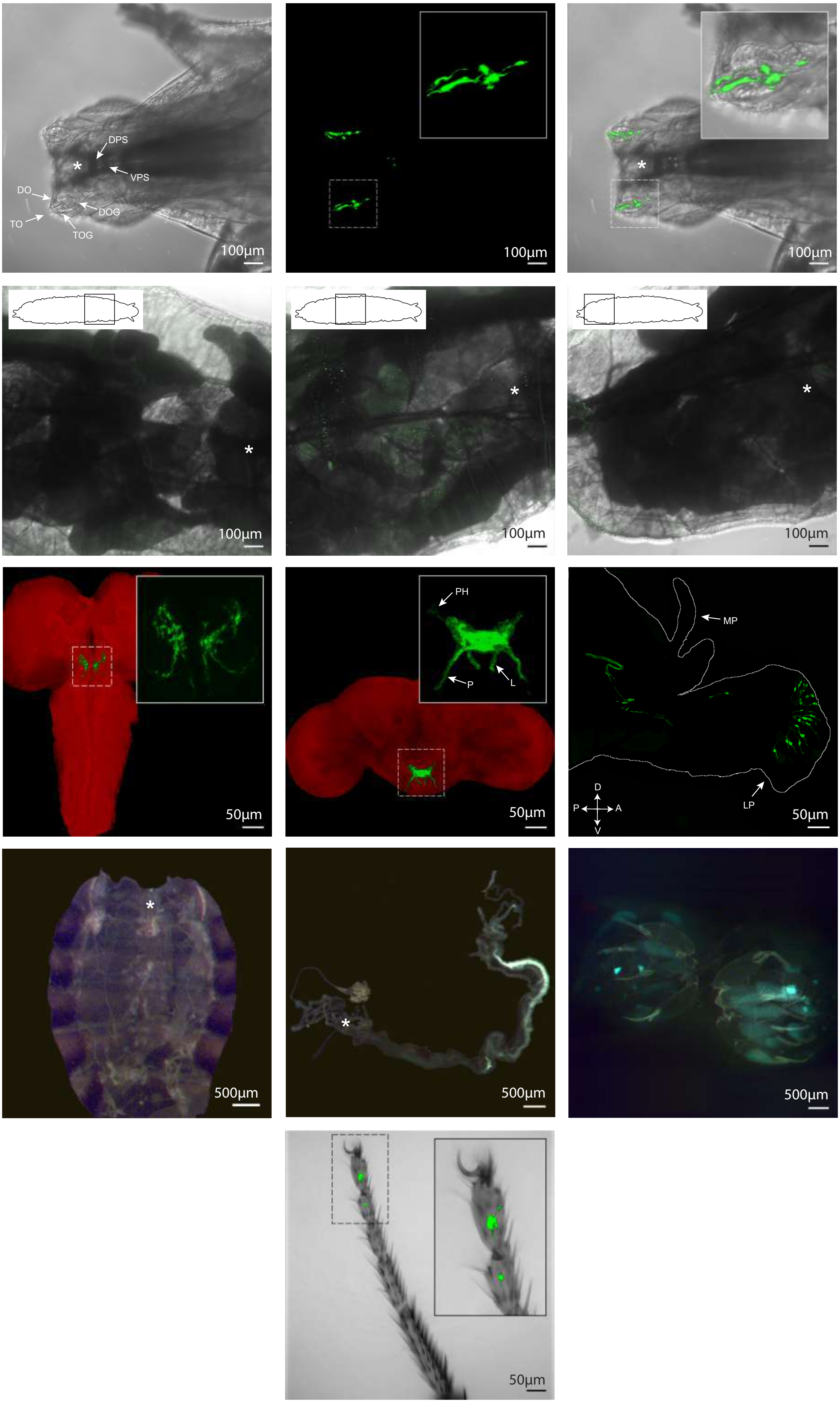

DPS
VPS
DO
DOG
TO
TOG
100µm
100µm
100µm
100µm
100µm
100µm
50µm
PH
P
L
50µm
MP
D
P
A
V
LP
50µm
500µm
500µm
500µm
50µm

Fig.S3: Gr66a-Gal4 expression pattern

Gr66a-Gal4/UAS-GFP larvae and adult females were dissected and the fluorescence was observed in larval taste neurons (first line pictures); projections in larval brain, adult taste neurons of the proboscis, projections in adult brains (third line pictures) and terminal tarsi of the legs (fifth line picture). We did not observe any fluorescence in larval gut (second line pictures), larval fat body (second line pictures), adult gut, adult carcass or adult ovaries (fourth line pictures). TO for Terminal Organ; DO for Dorsal Organ; TOG for Terminal Organ Ganglion; DOG for Dorsal Organ Ganglion; DPS for Dorsal Pharyngeal Sensory organ; VPS for Ventral Pharyngeal Sensory organ. P for axons emanating from neurons in the Proboscis; PH for axons emanating from neurons in the Pharynx; L for axons emanating from neurons in the Legs. MP for Maxillary Palp; LP for Labellum-Proboscis. The * defines the anterior of the portion shown. The boxes within pictures are magnifications of the area delimited by the dashed line. D, V, P and A for Dorsal, Ventral, Posterior and Anterior.

A

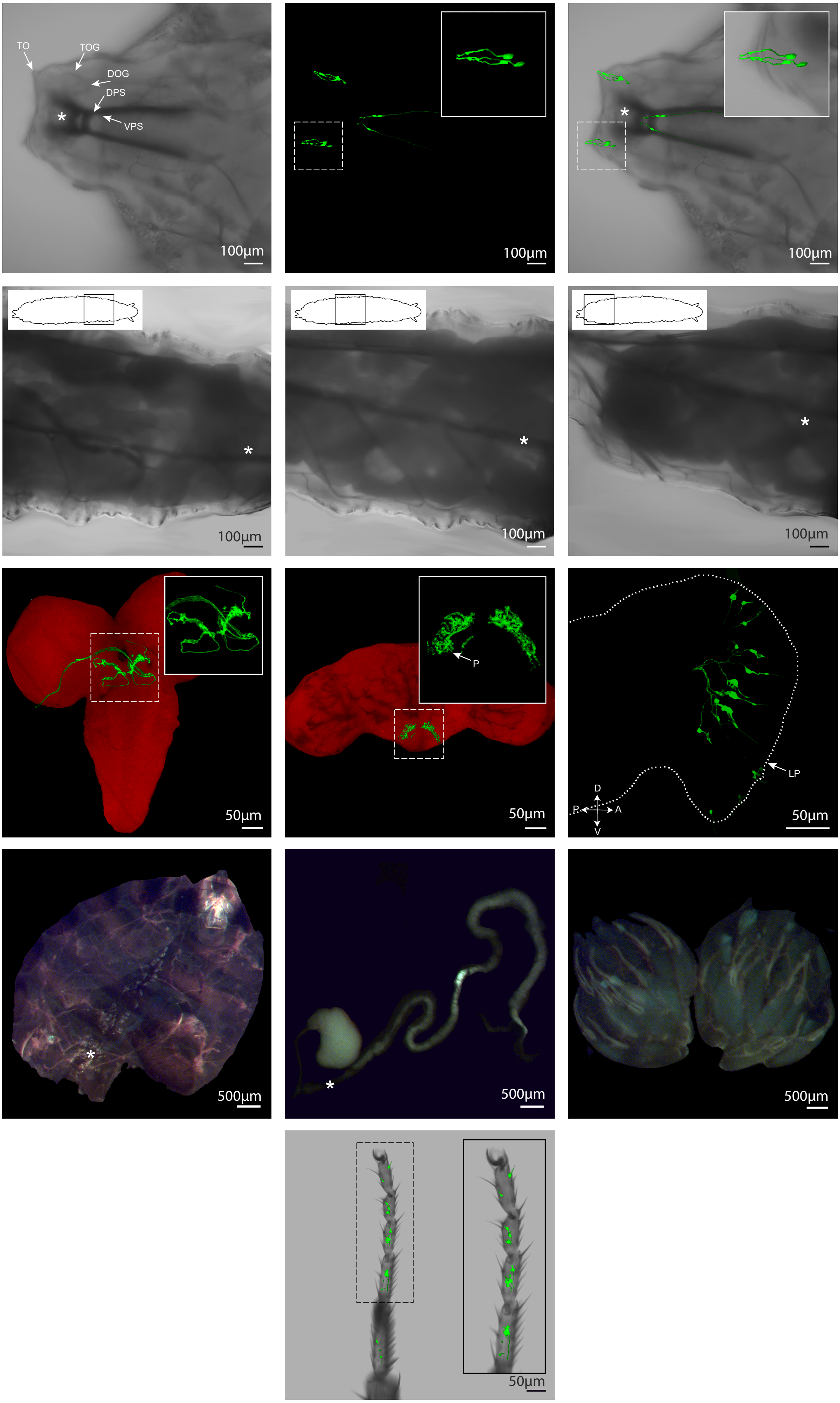
TO
TOG
DOG
DPS
VPS
100µm
100µm
100µm
100µm
100µm
100µm
P
LP
D
P
A
V
50µm
50µm
50µm
500µm
500µm
500µm
50µm

Fig. S4: **Ppk23-Gal4 expression pattern**

Ppk23-Gal4/UAS-GFP larvae and adult females were dissected and the fluorescence was observed in larval taste neurons, (first line pictures), projections in larval brain, adult taste neurons of the proboscis, projections in adult brains (third line pictures) and terminal tarsi of the legs (fifth line picture). We did not observe any fluorescence in larval gut (second line pictures), larval fat body (second line pictures), adult gut, adult carcass or adult ovaries (fourth line pictures). TO for Terminal Organ; TOG for Terminal Organ Ganglion; DOG for Dorsal Organ Ganglion; DPS for Dorsal Pharyngeal Sensory organ; VPS for Ventral Pharyngeal Sensory organ. P for axons emanating from neurons in the Proboscis. LP for Labellum-Proboscis. The * defines the anterior of the portion shown. The boxes within pictures are magnifications of the area delimited by the dashed line. D, V, P and A for Dorsal, Ventral, Posterior and Anterior.

A

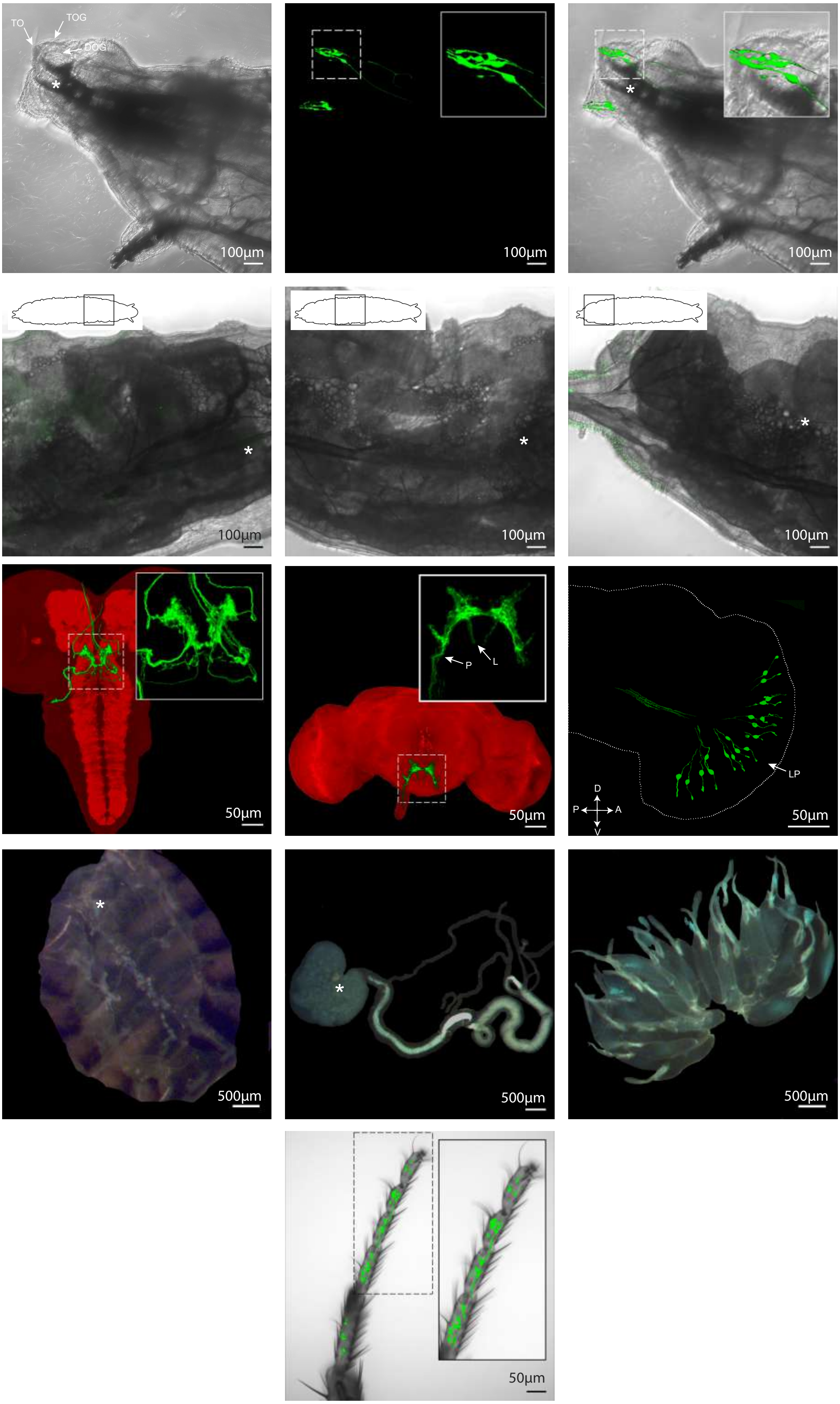
TO
TOG
DOG
100µm
100µm
100µm
100µm
100µm
100µm
50µm
P
L
50µm
LP
D
P
A
V
50µm
500µm
500µm
500µm
50µm

Fig. S5: Gr66a-LexA; LexAop-Gal80; ppk23-Gal4/UAS-GFP expression pattern

Larvae and adult females were dissected and the fluorescence was observed in larval taste neurons, (first line pictures), projections in larval brain, adult taste neurons of the proboscis, projections in adult brains (third line pictures) and terminal tarsi of the legs (fifth line picture). We did not observe any fluorescence in larval gut (second line pictures), larval fat body (second line pictures), adult gut, adult carcass or adult ovaries (fourth line pictures). TO for Terminal Organ; TOG for Terminal Organ Ganglion; DOG for Dorsal Organ Ganglion. P for axons emanating from neurons in the Proboscis; L for axons emanating from neurons in the Legs. LP for Labellum-Proboscis. The * defines the anterior of the portion shown. The boxes within pictures are magnifications of the area delimited by the dashed line. D, V, P and A for Dorsal, Ventral, Posterior and Anterior.

A

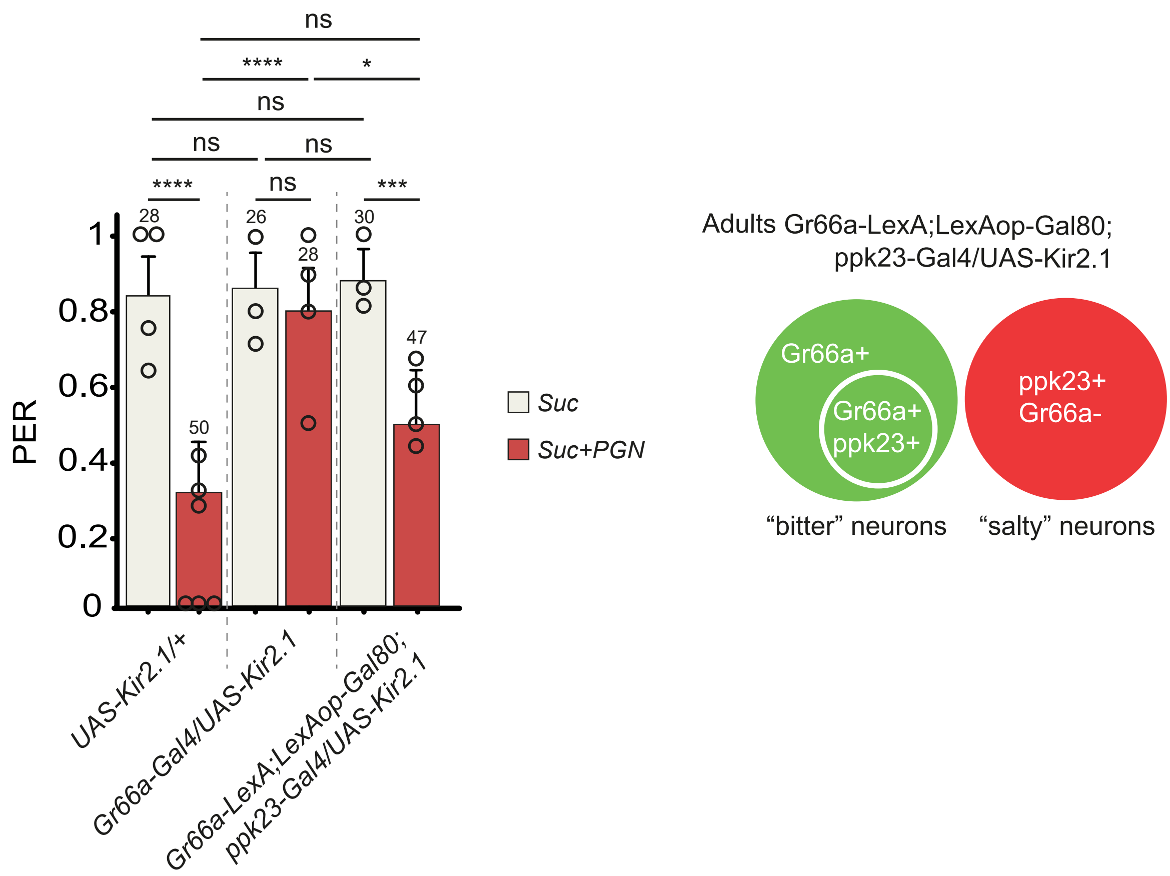

B

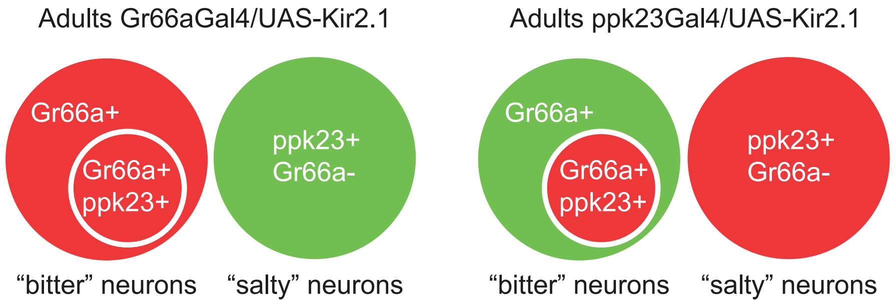

Fig.S6: Gr66a+/ppk23- and/or Gr66a+/ppk23+ neurons are sufficient to mediate PGN aversion

(**A**) Flies in which ppk23+/Gr66a- cells are silenced throughout development remain able to avoid PGN. PER index of flies to control solutions of sucrose and sucrose 1mM + PGN from *E. coli* K12 at 200µg/mL. The expression of LexAop-Gal80 in Gr66a+ cells antagonizes the activity of Gal4 in Gr66a+/ppk23+, thus preventing the expression of Kir2.1 in Gr66a+/ppk23+ neurons. Consequently, using ppk23-Gal4 as a driver, only ppk23+/Gr66a- neurons will be inactivated. The scheme represents in red the neurons inactivated by Kir2.1 and in green the active ones. (**B**) The scheme represents the result of driving the expression of Kir2.1 throughout development and without the intersectional genetic strategy in Gr66a+ or ppk23+ neurons, in red the neurons inactivated by Kir2.1 and in green the active ones. For (**A**) PER index is calculated as the percentage of flies tested that responded with a PER to the stimulation ± 95% CI. The number of tested flies (n) is indicated on top of each bar. For each condition, at least 3 groups with a minimum of 10 flies per group were used. ns indicates $p>0.05$, * indicates $p<0.05$, *** indicates $p<0.001$, **** indicates $p<0.0001$ two-sided Fisher Exact Test. Further details including raw data and exact p values can be found in the source data file.

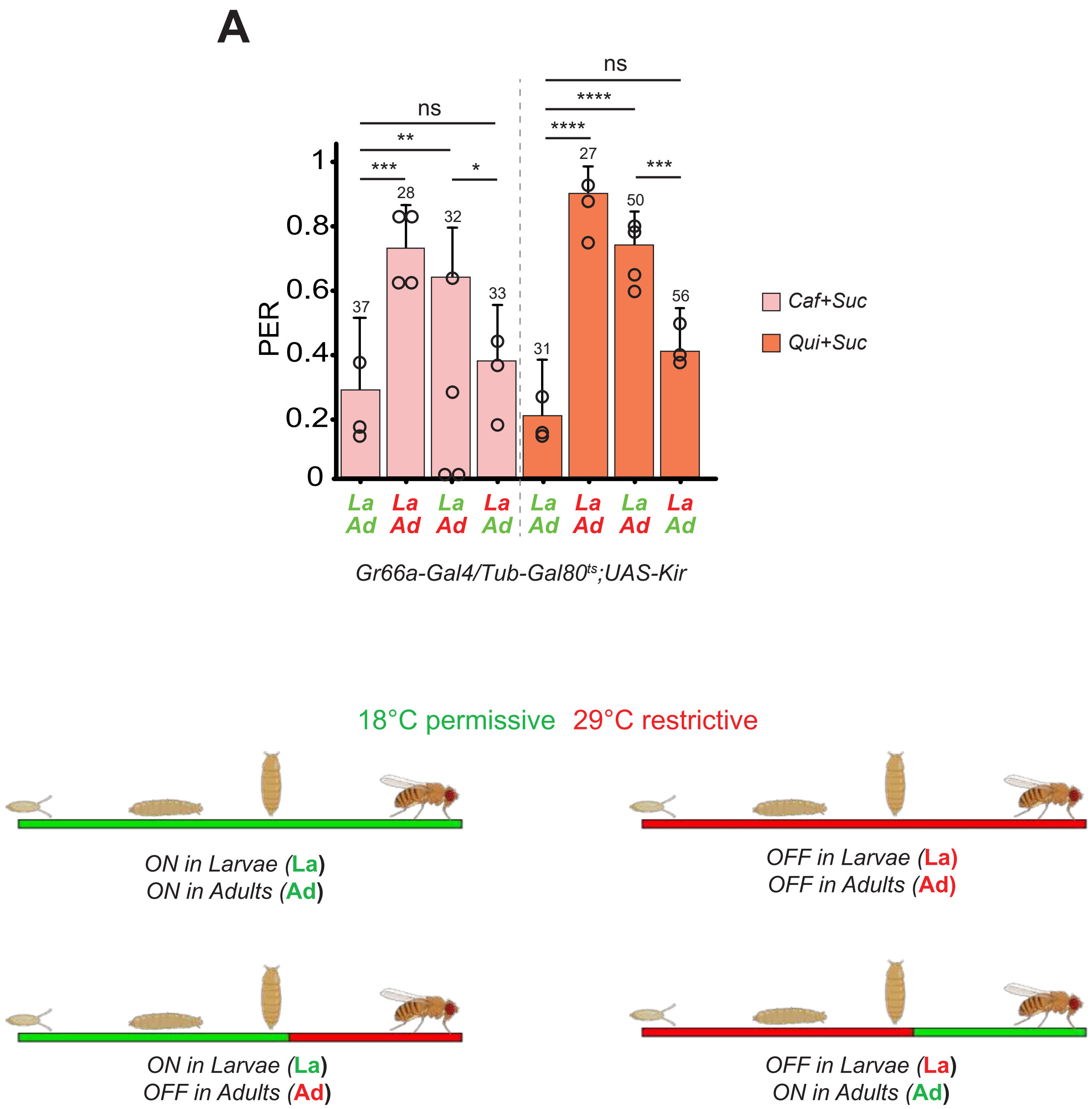

Fig.S7: Adult fly aversion to quinine and caffeine requires active adult Gr66a+ neurons

(**A**) Perception by the adult flies of sucrose 1mM + quinine 10mM and sucrose 1mM + caffeine 10mM required Gr66a+ neurons to be functional in the adult but not in larvae. As explained with the scheme, the ubiquitously expressed Tub-Gal80^{ts}, that inhibits the activity of Gal4, is temperature sensitive: it's active at 18°C and inactivated at 29°C, allowing the expression of UASKir2.1 and the consequent impairment of Gr66a+ or ppk23+ neuron activity. The PER index is calculated as the percentage of flies tested that responded with a PER to the stimulation ± 95% CI. The number of tested flies (n) is indicated on top of each bar. ns indicates p>0.05, * indicates p<0.05, ** indicates p<0.01, *** indicates p<0.001, **** indicates p<0.0001 two-sided Fisher Exact Test. Further details including raw data and exact p values can be found in the source data file.

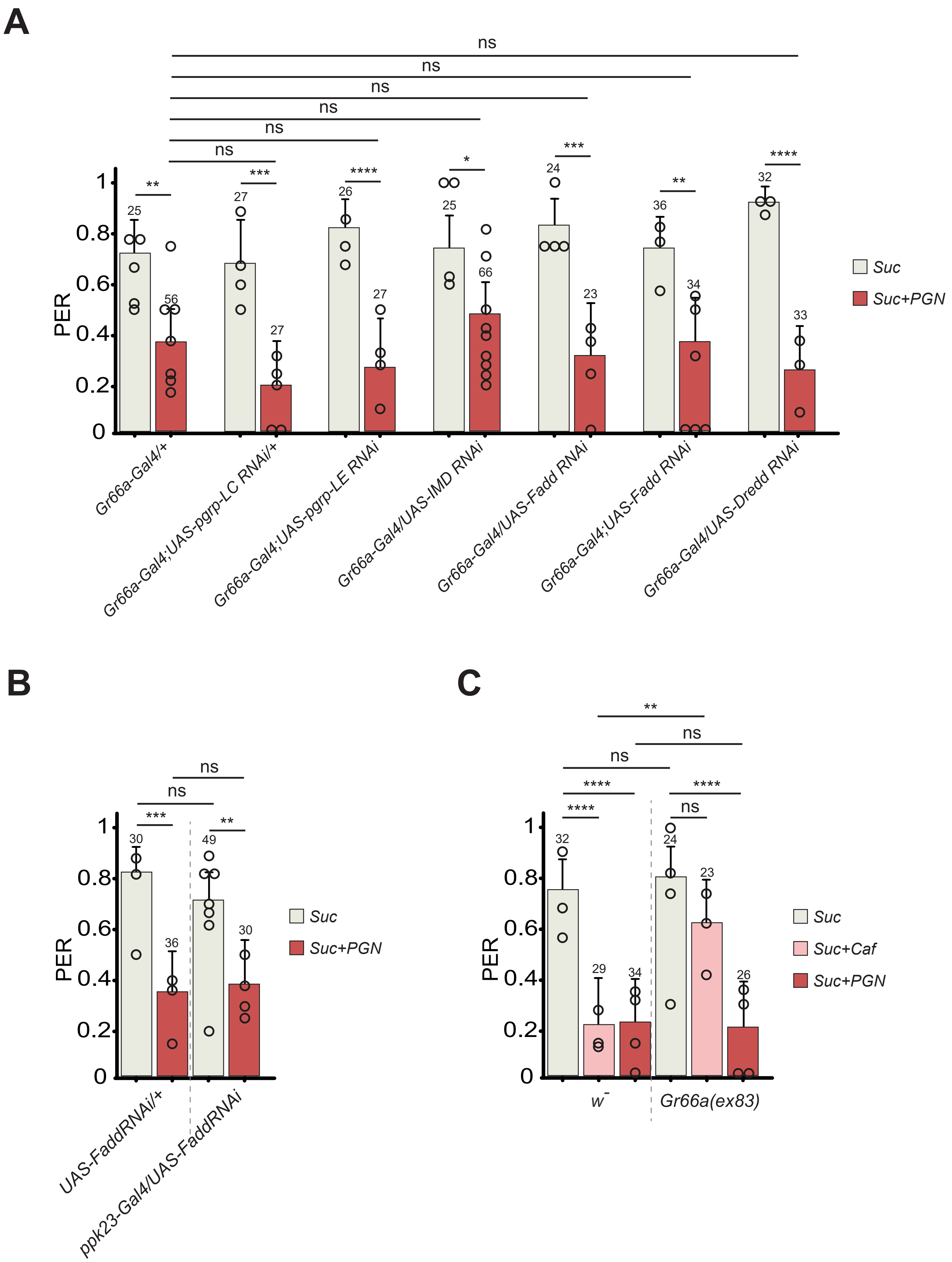
A
ns
ns
ns
ns
ns
ns
**
***
****
*
***
**
****
PER
1
0.8
0.6
0.4
0.2
0
25
56
27
27
26
27
25
66
24
23
36
34
32
33
Suc
Suc+PGN
Gr66a-Gal4/+
Gr66a-Gal4;UAS-pgrp-LC RNAi/+
Gr66a-Gal4;UAS-pgrp-LE RNAi
Gr66a-Gal4/UAS-IMD RNAi
Gr66a-Gal4/UAS-Fadd RNAi
Gr66a-Gal4;UAS-Fadd RNAi
Gr66a-Gal4/UAS-Dredd RNAi
B
ns
ns
***
**
PER
1
0.8
0.6
0.4
0.2
0
30
36
49
30
Suc
Suc+PGN
UAS-FaddRNAi/+
ppk23-Gal4/UAS-FaddRNAi
C
**
ns
ns
****
****
****
ns
PER
1
0.8
0.6
0.4
0.2
0
32
29
34
24
23
26
Suc
Suc+Caf
Suc+PGN
w-
Gr66a(ex83)

Fig.S8 : IMD pathway is not required in ppk23+ or Gr66a+ neurons to mediate aversion to PGN

(**A**) and (**B**) RNAi-mediated inactivation of different elements of the IMD pathway has no effect on the PGN induced PER suppression. (**A**) PER index of flies in which different elements of the IMD pathway are inactivated via RNAi in Gr66a+ neurons, to control solutions of sucrose and sucrose 1mM + PGN from *E. coli* K12 at 200µg/mL. (**B**) PER index of flies upon RNAi-mediated Fadd (UAS-Fadd RNAi) inactivation in ppk23+ cells, to control solutions of sucrose and sucrose + PGN from *E. coli* K12 at 200µg/mL. (**C**) The Gr66a receptor is not involved in PGN induced PER suppression, *Gr66a* mutants lose their aversion to caffeine but retain their aversion to PGN. PER index of fly mutant for the Gr66a receptor to control solutions of sucrose 1mM and sucrose 1mM + caffeine and to sucrose + PGN from *E. coli* K12 at 200µg/mL. For (**A**), (**B**) and (**C**) PER index is calculated as the percentage of flies tested that responded with a PER to the stimulation ± 95% CI. The number of tested flies (n) is indicated on top of each bar. For each condition, at least 3 groups with a minimum of 10 flies per group were used. ns indicates $p>0.05$, * indicates $p<0.05$, ** indicates $p<0.01$, *** indicates $p<0.001$, **** indicates $p<0.0001$ two-sided Fisher Exact Test. Further details including raw data and exact p values can be found in the source data file.

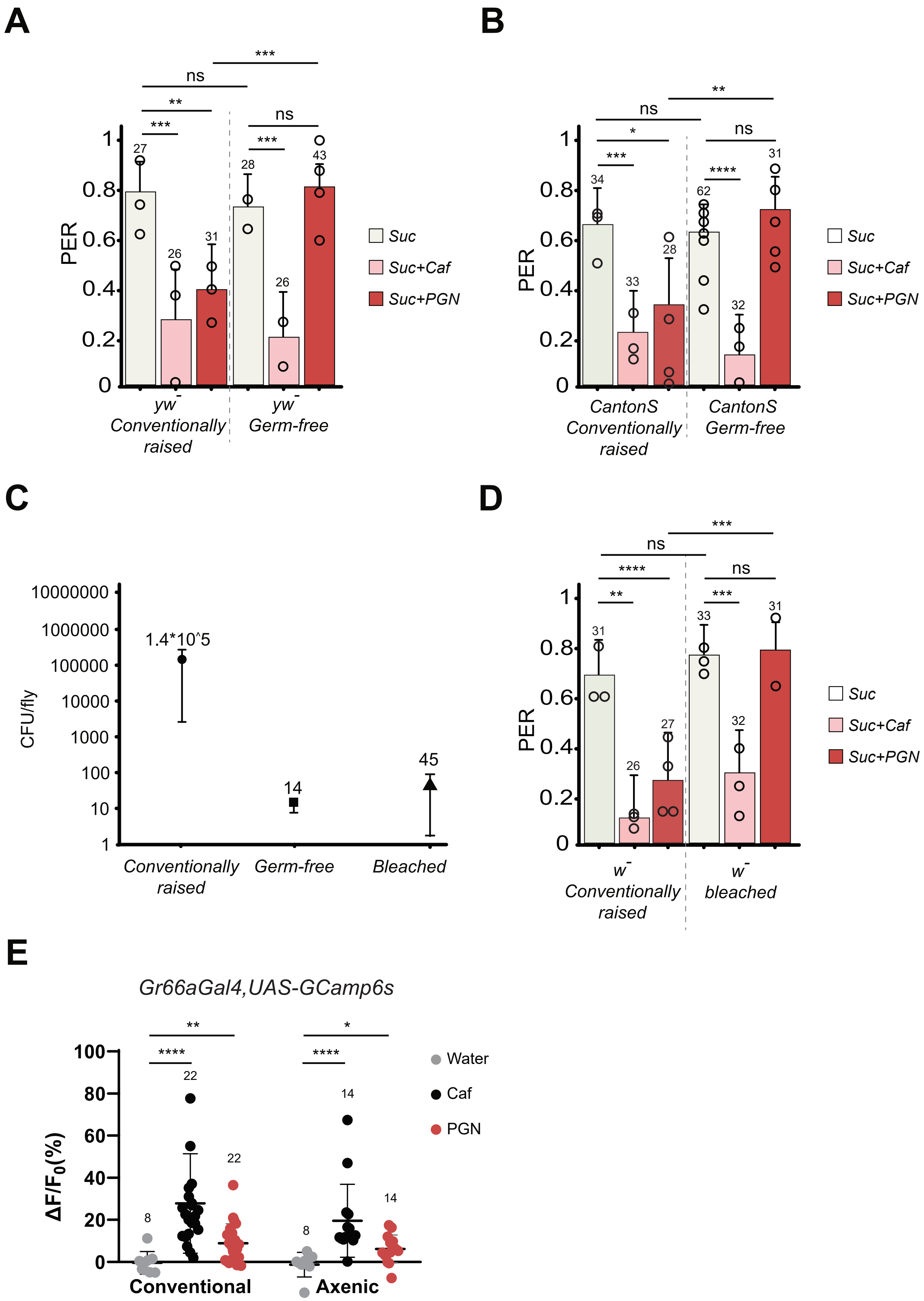
A
PER
Suc
Suc+Caf
Suc+PGN
yw-
Conventionally raised
yw-
Germ-free
B
CantonS
Conventionally raised
CantonS
Germ-free
C
CFU/fly
1.4*10^5
Conventionally raised
Germ-free
Bleached
D
w-
Conventionally raised
w-
bleached
E
Gr66aGal4,UAS-GCamp6s
ΔF/F0(%)
Water
Caf
PGN
Conventional
Axenic

Fig.S9: Germ free flies obtained without antibiotics do not perceive PGN as aversive

(A) and (**B**) Loss of aversion to PGN in axenic conditions is independent of genetic background. PER index of *yw*- (**A**) and CantonS (**B**) axenic flies to control solutions of sucrose 1mM and sucrose 1mM +caffeine 10mM and to sucrose 1mM + PGN from *E. coli* K12 at 200µg/mL. (**C**) and (**D**) the antibiotic treatment has no effect on the fly taste for PGN. (**C**) Bacterial load (CFU/fly) of *w*- flies reared under conventional conditions, germ-free with antibiotic treatment or sterilized by embryo bleaching. The data for conventionally raised and germ-free flies are the same as fig. 5A. (**D**) Flies emerged from bleached embryos retain the PGN induced PER suppression, PER index of *w*- flies sterilized by embryos bleaching to control solutions of sucrose 1mM and sucrose 1mM +caffeine 10mM and to sucrose 1mM + PGN from *E. coli* K12 at 200µg/mL. (**E**) Real-time calcium imaging using the calcium indicator GCaMP6s to reflect the *in vivo* neuronal activity of Gr66a+ neurons (Gr66a-Gal4, UAS-GCaMP6s) in adult brains of conventionally raised and germ-free flies whose proboscis has been stimulated with PGN. (**E**) Averaged fluorescence intensity of positive peaks ± SEM for conventionally raised (n= 22 flies) and germ-free flies (n= 14 flies) in response to caffeine 10mM and peptidoglycan from *E. coli* K12 at 200µg/mL. For (**A**),

(B) and (**D**) PER index is calculated as the percentage of flies tested that responded with a PER to the stimulation ± 95% CI. The number of tested flies (n) is indicated on top of each bar. For each condition, at least 3 groups with a minimum of 10 flies per group were used. ns indicates p>0.05, * indicates p<0.05, ** indicates p<0.01, *** indicates p<0.001, **** indicates p<0.0001 two-sided Fisher Exact Test. In (**E**), * indicates p<0.05, ** indicates p<0.001, **** indicates p<0.00001 non-parametric t-test, two-tailed Mann-Whitney test. Further details including raw data and exact p values can be found in the source data file.

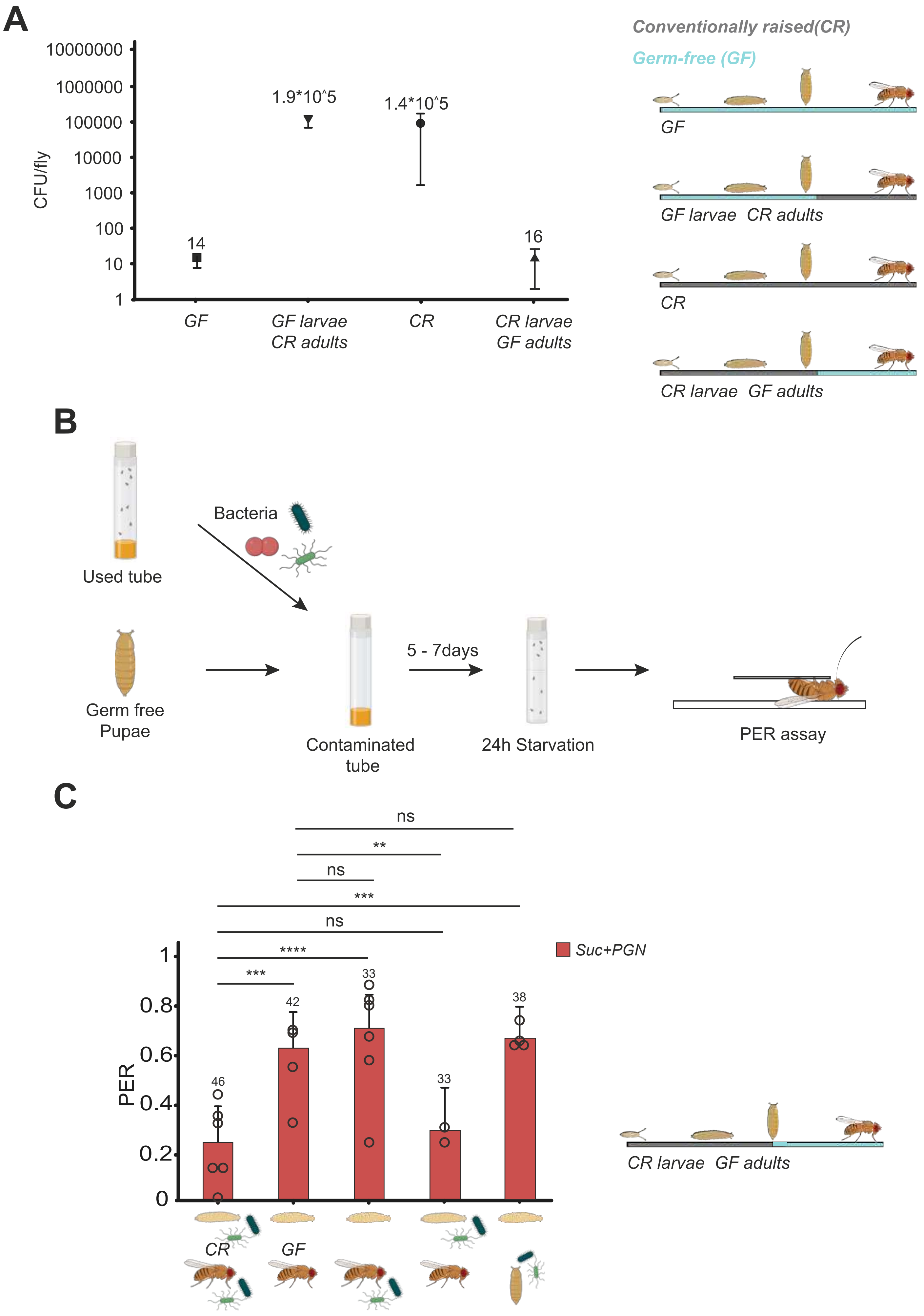
A
CFU/fly
10000000
1000000
100000
10000
1000
100
10
1
1.9*10^5
1.4*10^5
14
16
GF
GF larvae
CR adults
CR
CR larvae
GF adults
Conventionally raised(CR)
Germ-free (GF)
GF
GF larvae CR adults
CR
CR larvae GF adults
B
Used tube
Bacteria
Germ free
Pupae
Contaminated
tube
5 - 7days
24h Starvation
PER assay
C
ns
**
ns
***
ns
****
***
PER
1
0.8
0.6
0.4
0.2
0
46
42
33
33
38
Suc+PGN
CR
GF
CR larvae GF adults

Fig.S10 : Generating germ free flies

(**A**) Bacterial load (CFU/fly) of *w-* flies reared under conventional conditions (grey period), germ-free (blue period) or shifted between the two upon pupation. The data for conventionally raised and germ-free flies are the same as fig. 5A. (**B**) Graphical representation of the protocol for pupae contamination. *yw-* flies are reared under conventional conditions, after a few days the fly tube is filled with Luria Bertani's liquid medium and used as an inoculum to start a bacterial culture. The culture is then diluted to OD1 and used to soak filter paper placed over the fly culture medium in a new tube. The plug containing the germ-free pupae is dipped into the OD1 bacterial solution and used to close the previously prepared contaminated tube. (**C**) Contamination from the pupal stage is not sufficient to elicit PGN induced PER suppression. PER index of flies, that developed as germ-free larvae and were contaminated at the pupal stage, to sucrose 1mM + PGN from *E. coli* K12 at 200µg/mL. The data for flies raised in conventional conditions, germ free and for those switched between the two are the same as in Fig.6B. For (**C**) PER index is calculated as the percentage of flies tested that responded with a PER to the stimulation ± 95% CI. The number of tested flies (n) is indicated on top of each bar. For each condition, at least 3 groups with a minimum of 10 flies per group were used. ns indicates $p>0.05$, ** indicates $p<0.01$, *** indicates $p<0.001$, **** indicates $p<0.0001$ two-sided Fisher Exact Test. Further details including raw data and exact p values can be found in the source data file.

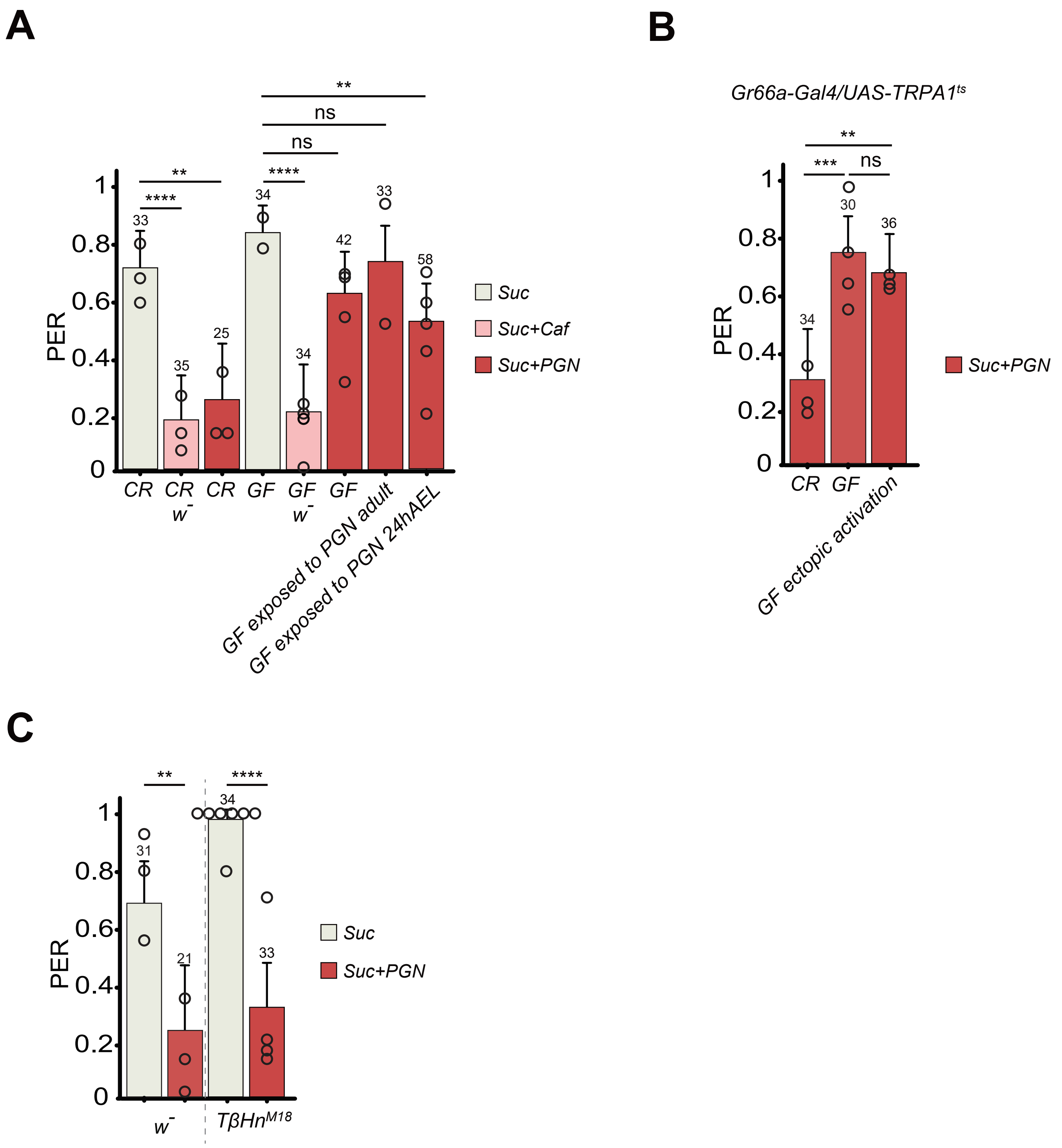

Fig.S11: Larval exposure to PGN is not sufficient to make germ free flies responsive to PGN

(**A**) PER index of germ-free flies exposed to PGN (DAP-type) as a larva or as adult, to sucrose 1mM + PGN from *E. coli* K12 at 200µg/mL. The data for germ-free flies (GF) are the same as in Fig.5B. (**B**) Activating ectopically Gr66a+ neurons during the larval life in germ-free (GF) conditions is not sufficient to obtain adults reacting to PGN. PER index of animals with the UAS-TRPA1^{ts} construction under the control of the Gr66a-Gal4 driver to control sucrose solution and to sucrose 1mM + PGN from *E. coli* K12 at 200µg/mL. Larvae and adults were raised at 18°C on classical media (CR) or germ-free media (GF) or on germ-free media with larvae at 29°C and adults at 18°C so the ectopic activation of Gr66a+ neurons is only triggered during the larval life. (**C**) Octopamine is not involved in PGN induced PER suppression. PER index of *TβH* mutant flies unable to synthetize octopamine, to control sucrose solution and to sucrose 1mM + PGN from *E. coli* K12 at 200µg/mL. For (**A**), (**B**) and (**C**), PER index is calculated as the percentage of flies tested that responded with a PER to the stimulation ± 95% CI. The number of tested flies (n) is indicated on top of each bar. For each condition, at least 3 groups with a minimum of 10 flies per group were used. ns indicates p>0.05, ** indicates p<0.01, **** indicates p<0.0001 two-sided Fisher Exact Test. Further details including raw data and exact p values can be found in the source data file.
